# *stk*: A python toolkit for supramolecular assembly

**DOI:** 10.1002/jcc.25377

**Published:** 2018-09-24

**Authors:** Lukas Turcani, Enrico Berardo, Kim E. Jelfs

**Affiliations:** ^1^ Department of Chemistry Imperial College London South Kensington SW7 2AZ London

**Keywords:** python, high‐throughput screening, supramolecular assembly, materials design, supramolecular chemistry

## Abstract

A tool for the automated assembly, molecular optimization and property calculation of supramolecular materials is presented. *stk* is a modular, extensible and open‐source Python library that provides a simple Python API and integration with third party computational codes. *stk* currently supports the construction of linear polymers, small linear oligomers, organic cages in multiple topologies and covalent organic frameworks (COFs) in multiple framework topologies, but is designed to be easy to extend to new, unrelated, supramolecules or new topologies. Extension to metal–organic frameworks (MOFs), metallocycles or supramolecules, such as catenanes, would be straightforward. Through integration with third party codes, *stk* offers the user the opportunity to explore the potential energy landscape of the assembled supramolecule and then calculate the supramolecule's structural features and properties. *stk* provides support for high‐throughput screening of large batches of supramolecules at a time. The source code of the program can be found at https://github.com/supramolecular-toolkit/stk. © 2018 The Authors. Journal of Computational Chemistry published by Wiley Periodicals, Inc.

## Introduction

In recent years, computational modeling has received increasing interest as a tool for both supporting and accelerating materials discovery. Molecular simulations have long been employed to offer insights by supplementing or rationalizing experimental data. However, computational screening is increasingly becoming a choice for the initial screening of large sets of materials, before any attempts at synthetic realization.^1^, ^2^, ^3^, ^4^, ^5^, ^6^, ^7^, ^8^, ^9^ This screening can not only identify the most promising targets for synthesis, but also narrow down the enormous phase for hypothetical materials to the most promising regions. In addition to the benefits of producing specific experimental targets, the materials modeling community is further making efforts to build and curate open‐source depositories of structures and properties of materials, to provide the data to make informed choices on material selection. Examples of initiatives in this area are the Materials Project,^10^ for high‐throughput computational screening of inorganic materials as part of the Materials Genome Initiative, and the Novel Materials Discovery (NOMAD) laboratory, a European Centre for Excellence,^11^ that curates a large database of computational materials data. Both of these initiatives are mostly oriented towards inorganic, solid‐state structures, rather than supramolecular materials.

Supramolecular materials result from the self‐assembly of a number of smaller molecular components, and can include molecular systems up to a large degree of complexity, such as macrocycles, organic cages, catenanes, rotaxanes, knots and molecular machines. Increasing the number of dimensions of these organic materials leads to linear polymers (one‐dimensional), and periodic materials such as covalent organic frameworks (COFs), which can be two‐, or three‐dimensional or amorphous polymeric systems (three‐dimensional). The inclusion of metal atoms in the materials leads to additional systems, such as metallocycles, metal–organic polyhedra (MOPs) and the highly topical metal–organic frameworks (MOFs). Beyond the inherent beauty of many of these structures, supramolecular materials have shown potential in a broad range of applications,^12^, ^13^, ^14^, ^15^, ^16^ including, but not limited to, sensing, molecular separation, catalysis, encapsulation and as electronic or photonic devices.

Nowadays, thanks to the multitude of freely available computational chemistry packages, such as ORCA,^17^ GAMESS,^18^ NWChem^19^ and MOPAC,^20^ automating the calculation of the properties of complex molecules can be a trivial task, whereas a challenging step involves the generation of realistic, and representative, structural models. To avoid this bottleneck in high‐throughput screening of supramolecular systems, research groups employ in‐house scripts to tackle the generation of specific molecular systems, but often their code is not made available to the wider scientific community, is hard to maintain, and difficult to generalize to a wider set of systems. A few programs are currently available for the generation of MOPs,^21^ biomolecules^22^, ^23^ and polymeric systems,^24^, ^25^, ^26^, ^27^ however, to the best of our knowledge, there is no open source package that supports both the assembly of complex supramolecules and which can be easily extended for use with new classes of supramolecular materials. With this in mind, we developed the ***s**upramolecular **t**ool**k**it* (*stk*), a Python library for general supramolecular assembly.

*stk* currently provides for the construction of complex supramolecules including molecular cages, linear polymers and covalent organic frameworks (COFs). Due to its modular design, it could be easily extended to support further, more exotic, supramolecular structures and even to structures containing metals, such as metallocycles, MOPs and MOFs. The key design principles of *stk* emphasize modularity and object‐oriented programing. Within *stk*, the underlying molecular representation is provided by RDKit,^28^ a cheminformatics Python library. Once an initial supramolecular structure is generated in *stk*, a multitude of operations are available to apply on the structure, for example, geometry optimization, conformer generation, molecular dynamics simulation, energy calculation or centroid calculation. The current *stk* version already directly interfaces with codes such as RDKit, Schrödinger's Macromodel^29^ and MOPAC, facilitating the interaction between them by providing a simple API for access to multiple features. MOPAC in particular provides for access to electronic properties such as total energies, ionization potential and electron affinity. *stk* can be readily extended to interface with other molecular simulation packages to allow for further desired operations to be carried out on the supramolecules. Finally, *stk* provides a simple API for the assembly, structure optimization and property calculation of molecules in batches and in parallel across available CPUs.

In this paper, we will first provide an overview of the overall structure of *stk*, then discuss the implementation via *stk* objects. Next, we will give an overview of how the supramolecular assembly from building blocks is conducted, before showcasing examples for the four supramolecular classes that are currently covered by *stk*; molecular cages (in 14 distinct topologies^30^), finite size linear polymers, small molecule oligomers and COFs (in 4 topologies). These examples will allow us to demonstrate some of the methods available for operations on any class of supramolecular material in *stk.* Further examples and a more thorough documentation can be found at https://lukasturcani.github.io/stk/docs/build/html/

## Software Overview

**Figure 1:**
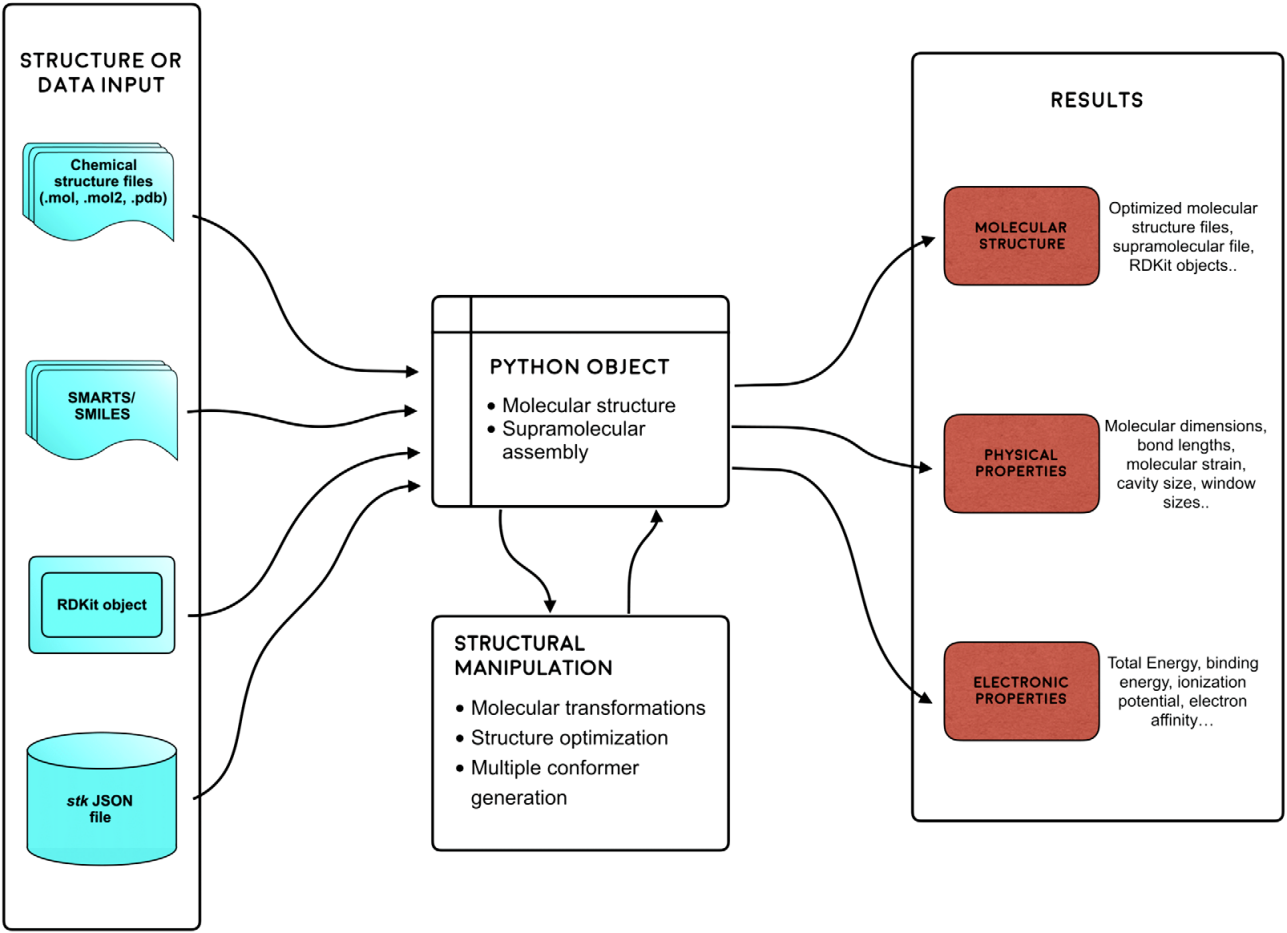
An outline of the key elements of the supramolecular toolkit software, *stk. stk* uses molecular structures as input, which can be stored in a variety of formats such as molecular structure files, SMILES strings or RDKit objects. These inputs are used to create *stk* Python objects of the molecules. The objects can be used to perform structural optimizations, conformer searches, molecular transformations, such as translations and rotations, and property calculation. Python objects can be output as molecular structure files, input files for computational codes or RDKit objects. Finally, *stk* molecular objects can be used to assemble supramolecules which are also represented as Python objects and can be used in the same way as those loaded into *stk* directly. [Color figure can be viewed at wileyonlinelibrary.com]

An overview of the functionality of *stk* is shown in Figure 1, and in the following sections the key features of the software are discussed.

### Input

The base input for *stk* are the molecules which act as building blocks for supramolecular structures. For example, an aldehyde and an amine molecule are used to form an imine molecular cage, or a dibromofluorene monomer is used to form a linear polyfluorene chain. These input building blocks can be provided to *stk* in a variety of formats, and, of course, this is easily extendable to new molecular structure file formats. Currently supported file types include MOL, MOL2, PDB and Macromodel MAE files. In addition, SMILES and SMARTS strings and RDKit molecule objects can alternatively be used as input, which allows *stk* to easily integrate with diverse workflows and existing scripts.

### Model construction

The assembly process for a given supramolecule is specified by defining a Python function within *stk.* The function is responsible for ensuring that the molecular building blocks are correctly placed and connected to produce the desired structure. It is often sufficient to define one such function for a large group of molecules. For example, after the general algorithm for assembling cages was defined, a total of 14 different cage topologies^30^ were added by simply specifying the coordinates of the vertices in a topology and defining which of these vertices are connected by edges. To make the definition of assembly functions easier, *stk* provides a variety of methods that manipulate the positions and orientations of building block molecules.

### Structure manipulation

After the building blocks have been assembled into a supramolecule, the resulting structure is unlikely to correspond to a minimum on the potential energy surface (PES). This is because building blocks are placed on a shape which corresponds to an idealized structure of the supramolecule, as defined by the assembly function. For example, the building blocks of a linear polymer are placed in a straight chain, even though the minimum energy structure may be twisted, bent or otherwise nonlinear. By defining more complex assembly functions, structures closer to the minimum of the PES may be generated. However, this can often result in duplicated effort, as structure optimization tools for many supramolecules already exist. As a result, structure optimization by *stk* is handled by providing integration with third party tools. For example, *stk* provides a function that writes the structure of a molecule into a Maestro structure file, writes a MacroModel input file, runs MacroModel, parses the output of MacroModel and updates the structure of the Python object with the result. This approach is easily duplicated with other third party software. However, this is not the only way structure manipulation can be implemented within *stk.* The only requirement for manipulation functions is to define a Python function which modifies the structure of the molecule, there are no requirements as to how it should accomplish this, be it invoking third party software or defining a custom algorithm. Finally, manipulation functions provided by *stk* will work with any molecule loaded into *stk*, be it an assembled supramolecule or a plain molecule loaded via the aforementioned input methods. This means *stk* can be used as a general Python API for interfacing with third party tools, as any molecular structure can be loaded into *stk* and a chosen manipulation function can be applied to it.

An advantage of our approach, is that *stk* does not force any particular global minimization method onto an assembled supramolecule. This is particularly important as different supramolecules will require vastly different procedures to generate realistic structures. Forcing an approach would result in loss of generality. For example, in our work with cage molecules, we found that first minimizing the distance between the bonds added during assembly, while keeping the remaining bond distances in the structure fixed, was key to producing realistic structures. This step is followed by an unconstrained geometry optimization, which in turn is followed by a conformer search, performed by simulated annealing. Each sampled structure is geometry optimized and the lowest energy conformer is used as the final cage structure. With simpler systems, *stk* can use RDKit's fast ETKDG algorithm^31^ as the manipulation function, which may provide reasonable structures much faster, especially for small molecules. Note that this discussion does not provide an exhaustive list of structure manipulation functions available within *stk*, as integration with other third party software such as MOPAC is also available.

### Property calculation

Once an *stk* molecule object is generated, methods for the calculation of a broad range of molecular properties become available. Due to the integration with RDKit, any functionality provided by this library is automatically included. This includes tools for drawing molecules, substructure searching, fingerprinting, molecular similarity and descriptor calculation. In addition to these tools, *stk* provides functions to calculate physical and electronic properties as well as perform energy calculations. Similar to the structure manipulation functions, property calculation functions may invoke third party software when necessary. However, regardless of how these functions are implemented, a key benefit of *stk* is that it allows the user to always remain in a Python environment.

### Output

In addition to the returned physical and electronic properties, the constructed molecule objects of supramolecules can be saved in a variety of formats, including MDL, Macromodel structure files, PDB, SMILES and InChI. *stk* objects can also be stored as JSON files, which can be reloaded to restore an *stk* object in a new Python session.

### Batch processing

*stk* provides support for high‐throughput screening by assembling, optimizing and analyzing large batches of supramolecules at a time via **Population** objects. Given a set of building blocks, a population of all possible supramolecules can be created by considering every combination. Alternatively, a random set of supramolecules can be produced by selecting building blocks at random. Finally, *stk* supports the generation of a “chemically diverse” population. In this case, half of the supramolecules in the population are generated by randomly selecting building blocks from the set. Each random supramolecule is then used to produce a “chemically diverse” analog. This is achieved by finding the building blocks in the set which are least similar to those making up the random supramolecule. The chemical similarity is calculated using Dice similarity on the Morgan fingerprints generated by RDKit.

## *stk* Objects

**Figure 2:**
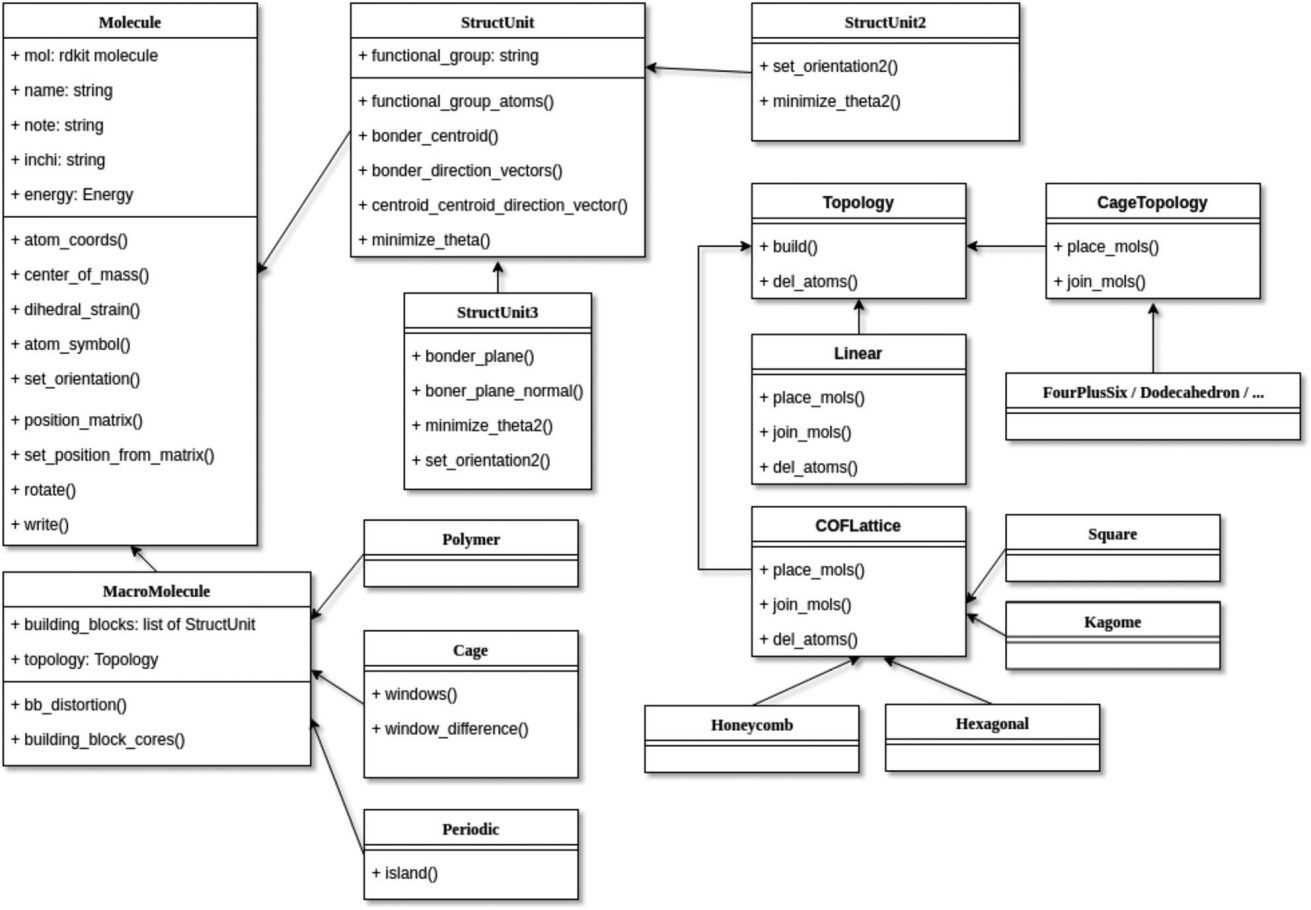
Class hierarchy of *stk.* Only a selection of key attributes and methods is shown for each class. The **Molecule** class serves as a base class for all classes which represent molecules. The **StructUnit** class serves as a base class for all classes which represent building blocks. **StructUnit2** represents building blocks with two functional groups while **StructUnit3** represents building blocks with three or more functional groups. The **MacroMolecule** class represents assembled supramolecules. It serves as a base class for classes which represent specific supramolecule types, such as **Polymer, Cage** and **Periodic**. The **Topology** class is a base class for classes which implement a supramolecular topology via an assembly function. The **Molecule, MacroMolecule, Topology, CageTopology** and **COFLattice** classes are used as abstract base classes and are not used to initialize objects directly.

In this section, we provide an overview of the classes which make up *stk* and their interactions. A summary is shown in Figure 2. This is followed by an explanation of how instances of these classes are used to assemble specific supramolecules.

### Molecule

To work with molecules, *stk* provides a hierarchy of classes, as outlined in Figure 2. The base class used for all molecule objects is called **Molecule**. It is inherited by all classes which describe molecules, be it building blocks or supramolecules. It includes methods for the calculation of structural properties, such as molecular size and centre of mass, and methods for manipulating a molecule's position, such as applying rotations and translations and aligning any arbitrary component of the molecule with any arbitrary axis. Finally, **Molecule** includes tools for the calculation of electronic properties, such as energy, ionization potential and electron affinity. For a complete list of properties that can be calculated, the reader is referred to the *stk* documentation.^32^ This class is easily extended with additional methods to calculate molecular properties or perform new operations. This can be done by interfacing with suitable computational chemistry codes when necessary.

### StructUnit

The second basic class, **StructUnit**, represents the building blocks of the supramolecules. While supporting all operations of the **Molecule** class, additional operations which may be necessary for constructing supramolecules are added, for example, the method “bonder_centroid” returns the centroid of all the atoms in the building block which form new bonds during the supramolecular assembly process. This class is extended by the **StructUnit2** and **StructUnit3** subclasses. **StructUnit2** adds operations for the alignment of building blocks with 2 functional groups (di‐topic) when assembling a supramolecule. Equivalently, **StructUnit3** adds operations for 3 (tri‐topic) or more functional groups. The user can specify which building block conformers to use during construction, to provide a greater degree of control over the final structure of the assembled supramolecule.

### MacroMolecule

The **MacroMolecule** class represents assembled supramolecules; as with the **Molecule** class, this class serves as an abstract base class and is not used to initialize objects directly. Instead, a new subclass is made for each supramolecule, holding any additional operations which that supramolecule may require. For example, the **Polymer, Periodic** and **Cage** classes represent finite size polymers (and short oligomers), periodic materials and covalent organic cages, respectively. As *stk* supports storing multiple conformers for each supramolecule, the user can specify which conformer needs to be used when calculating molecular properties. This allows the user to easily evaluate how molecular properties change between conformations, for example when searching for the structure which best matches experimental properties.

### Topology

The topology of a supramolecule defines the underlying connectivity of the building blocks in the final supramolecule, and is a feature that does not change upon deformation. For example, a molecular cage can have a tetrahedral, **Tri^4^Di^6^**, or cubic, **Tri^8^Di^12^**, topology, among many others. Similarly, a polymer may be linear or branched. The class **Topology** is used in *stk* to assemble the supramolecules from constituent building blocks into a defined topology. Whenever a supramolecule object is created, a topology object must be provided so that a molecule with the correct structure is formed. For example, the **Linear** topology defines a linear polymer and so an instance of the **Linear** class will define the repeating unit as well as the number of repeating units. A small number of repeating units can generate a short oligomer. This instance is then provided to the initializer when constructing a **Polymer** object. *stk* can be extended with new topology classes to add support for the construction of new types supramolecules, such as MOFs or rotaxanes, or new topologies of existing supramolecules. Though there is no inherent limit to the classes of molecules which can be added, more complex supramolecules will require contributors to provide more complex assembly functions.

## Supramolecular Assembly Process

Next we will give a descriptive overview of the general procedure that is carried out to take molecular building blocks and arrange them into supramolecular structures; in the following section, we will then illustrate how this is applied to four specific classes of supramolecular materials. When assembling supramolecules in *stk*, the assembly process requires two independent pieces of information. First, the building blocks need to be supplied, and these can be either molecules or molecular fragments. As mentioned in the software overview, the building blocks may be provided in a variety of input formats. Second, the topology needs to be defined, as this establishes how the building blocks are connected together to form the supramolecule. At a minimum, the topology must be defined as a child class of **Topology** and contain the assembly function as a method. Topologies must be defined ahead of time, and then when the user runs *stk*, they need only to state which topology to use and the appropriate function will be executed. Assembly can also be performed in parallelized batches by providing the *stk* **Population** class with groups of building blocks and topologies.

**Figure 3:**
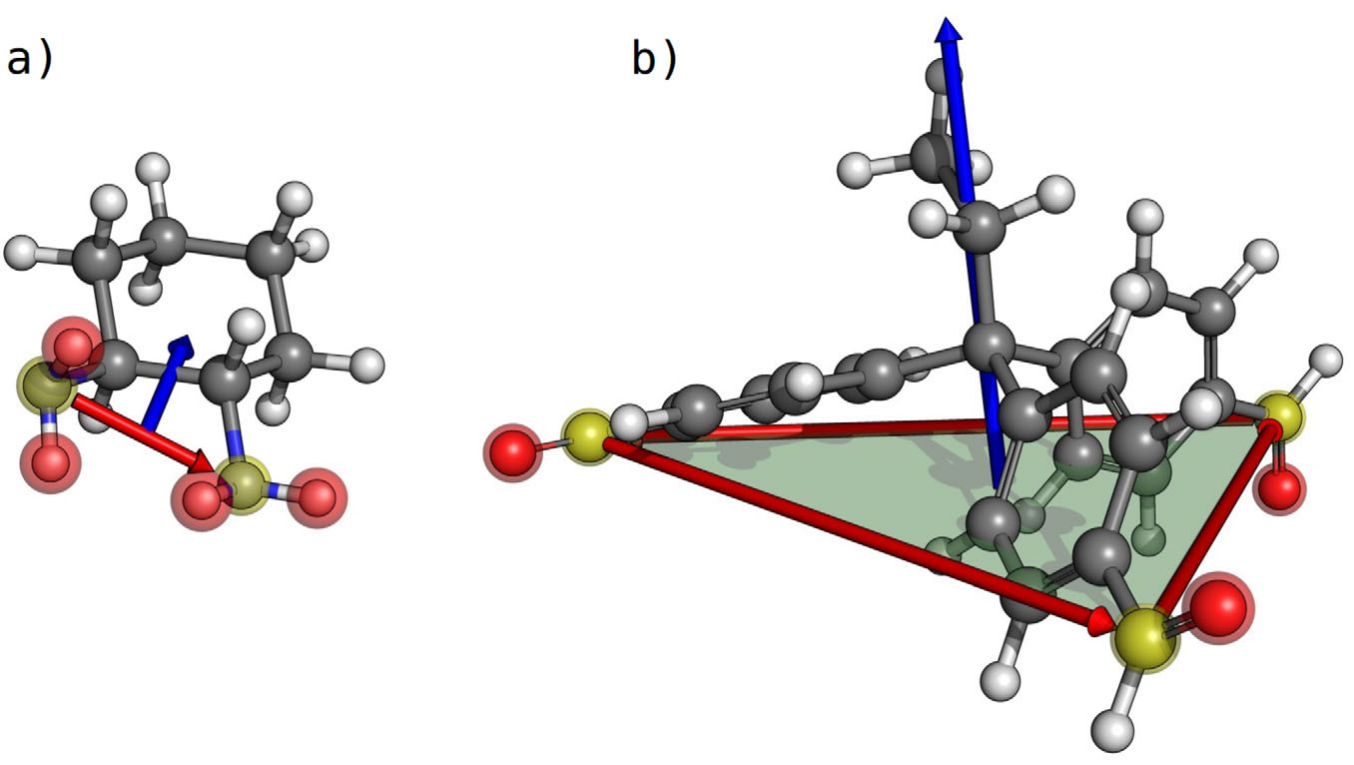
Functional group identification and vectors used for alignment during supramolecular assembly, shown on a) a diamine and b) a trialdehyde building block. The atoms directly involved in bond formation, “bonder atoms,” are highlighted in yellow; these are the nitrogen atom of the amine group and the carbon atom of the aldehyde group. The atoms that need to be removed upon bond formation, “deleter atoms,” are highlighted in red; these are the hydrogens of the amine group and the oxygen atom of the aldehyde group. The bonder–bonder direction vectors running between the bonder atoms are shown with red arrows. The centroid–centroid direction vectors, running from the centroid of the bonder atoms in each building block towards the centroid of the molecule, are shown in blue. For the trialdehyde, the plane used to align molecules with 3 or more functional groups is shown in green. [Color figure can be viewed at wileyonlinelibrary.com]

After the building blocks have been loaded into **StructUnit** instances, *stk* identifies the atoms that will be directly involved in bond formation during assembly. To do this, *stk* has a database of functional groups. A “functional group” in *stk* consists of a group of atoms in a building block that are known to be directly involved in the assembly process. Functional groups in *stk* may match real, chemical functional groups, such as the —NH_2_ groups in amines and the —C(H)=O groups in aldehydes, but this is not a requirement. Thus, *stk* can form bonds between any arbitrary, user defined, set of atoms within the building blocks. The functional group identification for an example pair of building blocks is shown in Figure 3. Atoms which form bonds during assembly are called “bonder atoms” (highlighted in yellow in Fig. 3), and there is also the option for atoms from a functional group to be removed from the assembled supramolecule, the latter are called “deleter atoms” (highlighted in red in Fig. 3). For example, if there is an amine building block forming a supramolecule by imine condensation, the nitrogen atoms of an amine group are the “bonder atoms” and the two hydrogen atoms of each amine group are the “deleter atoms.” When providing a building block, the user may specify the name of the functional group to be used for assembly, otherwise *stk* will attempt to deduce it from the file name by checking if the name of a functional group is present. This framework allows users to mimic real chemical reactions during supramolecular assembly, such as imine condensation, which is beneficial when access to databases of chemical precursors is available, as building blocks can be used directly without any modifications. However, because *stk* is not restricted to chemical functional groups, this approach integrates seamlessly with molecular fragments and can be used to produce any arbitrary bonding between building blocks.

To easily position and align building blocks on the topology of a supramolecule, *stk* defines a set of vectors passing through each building block, as shown in Figure 3. For building blocks with two functional groups, two vectors are defined. The first vector runs between the two bonder atoms of the two functional groups; this is known as the bonder–bonder direction vector (red in Fig. 3). This vector allows the building block to be aligned along the axes where the new bond is formed. The second vector runs from the centroid of the bonder atoms towards the centroid of the molecule; this is known as the centroid–centroid direction vector and is shown in blue in Figure 3. When rotating a molecule about the bonder–bonder direction vector, the centroid–centroid direction vector allows control over where the majority of the molecule's mass will be placed. This functionality is necessary to ensure structural control over the assembled supramolecules. For example, when defining the molecular cage assembly process it means that the majority of the atoms of a building block could be placed outside, rather than inside, the internal cavity. This is key to generating shape‐persistent molecular cage structures. For building blocks with three or more functional groups, the first three functional groups are selected and the vectors running between them are used to define a plane, shown in green for the trialdehyde in Figure 3. The normal to this plane is used for the alignment of these building blocks within the selected topology.

The procedure for the geometric arrangement of the building blocks into the supramolecule of desired topology is best understood through the elucidation of the specific material examples in the next section. However, in brief the procedure can be summarized as: (i) each building block is placed and aligned at the appropriate position and orientation for the given topology; (ii) bonds are formed between the bonder atoms, as specified by the topology function; (iii) the deleter atoms, if any, are removed and (iv) the structure is geometry optimized. This is performed by third party software integrated into *stk.* Currently supported techniques for structural optimization include forcefield optimization, conformational searching using a simulated annealing procedure with molecular dynamics simulations, or the ETKDG method implemented in RDKit.^31^

## Examples of Supported Materials

*stk* has the potential to construct a broad range of supramolecules in user defined topologies. The program already supports molecular cages and COFs in a range of topologies, linear polymers and short oligomers, including dimers. These materials, which differ substantially in their underlying construction, are now discussed as demonstrations of *stk.* Each of these four materials is of significant research interest for a range of applications including molecular separations, encapsulation, sensing, (photo)catalysis and electronic devices, as discussed in multiple reviews.^12^, ^13^, ^14^, ^15^, ^16^ *stk* will be extended to support more supramolecular material classes in the future, as one of its key design objectives is simple extensibility.

### Porous organic cages

#### Context

Porous organic cages belong to a class of porous materials that lack an extended three‐dimensional network of covalent bonds.^12^, ^13^, ^33^, ^34^, ^35^ Porous organic cages consist of molecules that have a cavity with the potential to host guests and multiple entry and exit routes to that cavity through molecular “windows.” Their modular nature allows for the potential of a “mix‐and‐match” strategy for property tuning^36^ and their solution processability also offers potential advantages, for example in membrane fabrication,^37^ over three‐dimensional framework materials, such as zeolites and MOFs. Porous organic cages have shown potential for a variety of applications, such as molecular separation,^38^ sensing^39^ and as porous liquids.^40^

Organic cages are often the product of reversible reactions via dynamic covalent chemistry (DCC) between a pair of building blocks. Among the most commonly applied reactions to date is imine condensation reaction between aldehyde and amine precursors.^35^ The organic cage molecules resulting from such DCC reactions can adopt one (or more) of a range of potential topologies, often relating in geometric shape to well known polyhedra such as tetrahedrons, cubes and dodecahedra.^30^ Control over which topology will form is one of the challenges in cage synthesis, as there are frequent instances of “emergent behavior,” whereby an unanticipated (and potentially undesirable) topology formed.^41^ Factors such as the number of reactive end groups, the geometry of monomers, solvent choice and processing conditions have been shown to play a role.^30^ There have been reports of organic cages containing up to a total of 3 constituent molecules, consisting of 1 tritopic building block and 2 ditopic building blocks.^40^, ^42^, ^43^

To precisely identify a cage topology, a nomenclature was recently developed, which identifies the number of each building blocks incorporated into a cage and the number of functional groups per building block.^30^ The general structure is $X_{p}^{m}Y^{n}$, where *X* and *Y* correspond to building blocks constituting a cage and will be **Di** for ditopic, **Tri** for tritopic and **Tet** for tetratopic building blocks. *X* and *Y* should be ordered such that the number of reactive end groups in *X* is always greater than or equal to *Y.* The subscripts *m* and *n* identify the number of times each building block can be found within the cage. The subscript *p* identifies how many building blocks of type *Y* connect two building blocks of type *X.* When *p* = 1, this subscript is optional. Using this nomenclature, cage topologies can be divided into four families, shown in Figures 7. These are the (**Tri** + **Di**), (**Tet + Di**), (**Tri + Tri**) and (**Tet + Tri**) families.

**Figure 4:**
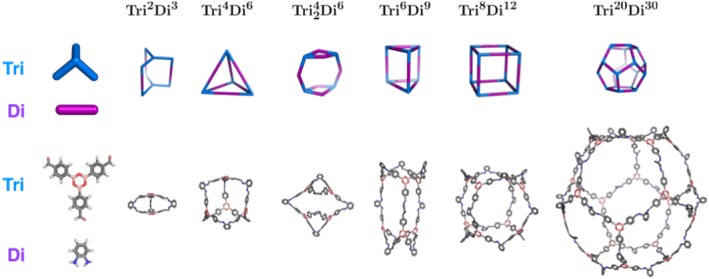
Cage topologies of the (**Tri + Di**) family that can be constructed by *stk.* The molecular building blocks are shown on the left and for the topologies, schematics of the topologies are shown above examples constructed and optimized with *stk* from different numbers of the molecular building blocks. Some of the topologies are highly strained for this particular building block combination. Hydrogen atoms are not shown in the topologies. [Color figure can be viewed at wileyonlinelibrary.com]

**Figure 5:**
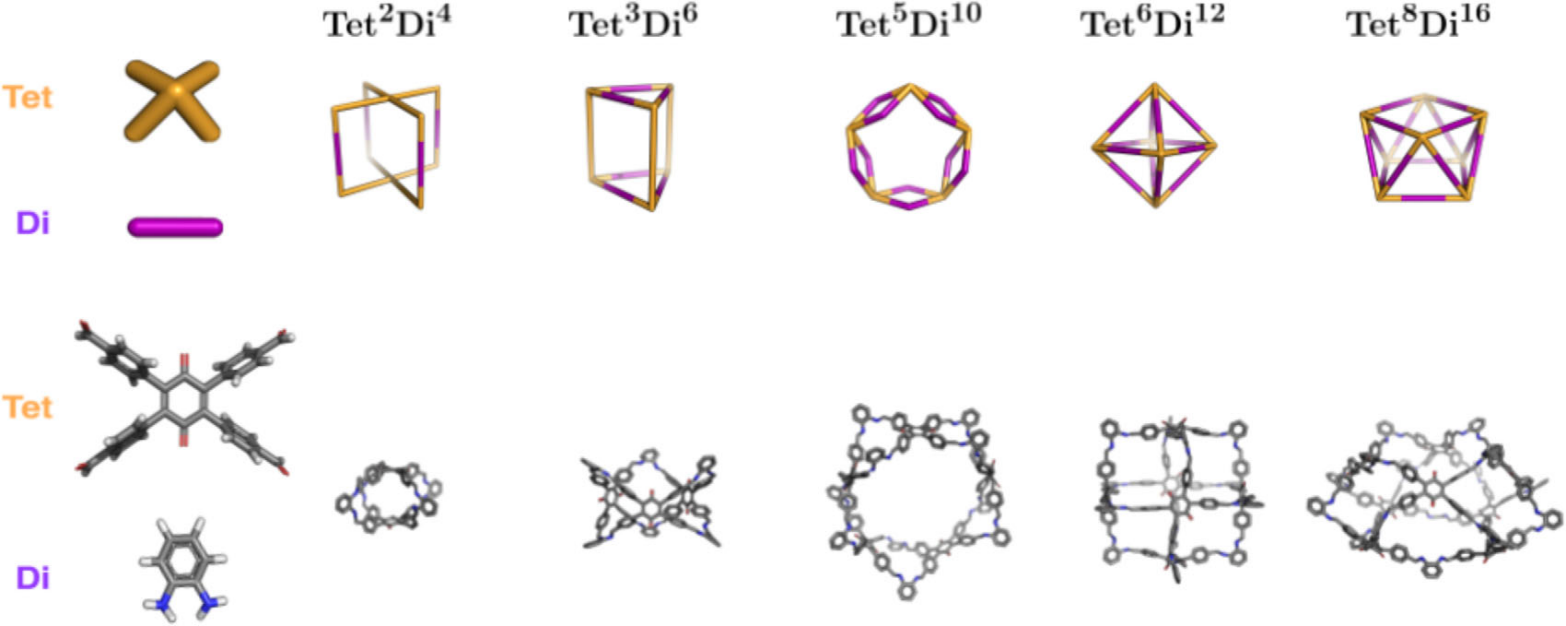
Cage topologies of the (**Tet + Di**) family that can be constructed by *stk.* The molecular building blocks are shown on the left and for the topologies, schematics of the topologies are shown above examples constructed and optimized with *stk* from different numbers of the molecular building blocks. Some of the topologies are highly strained for this particular building block combination. Hydrogen atoms are not shown in the topologies. [Color figure can be viewed at wileyonlinelibrary.com]

**Figure 6:**
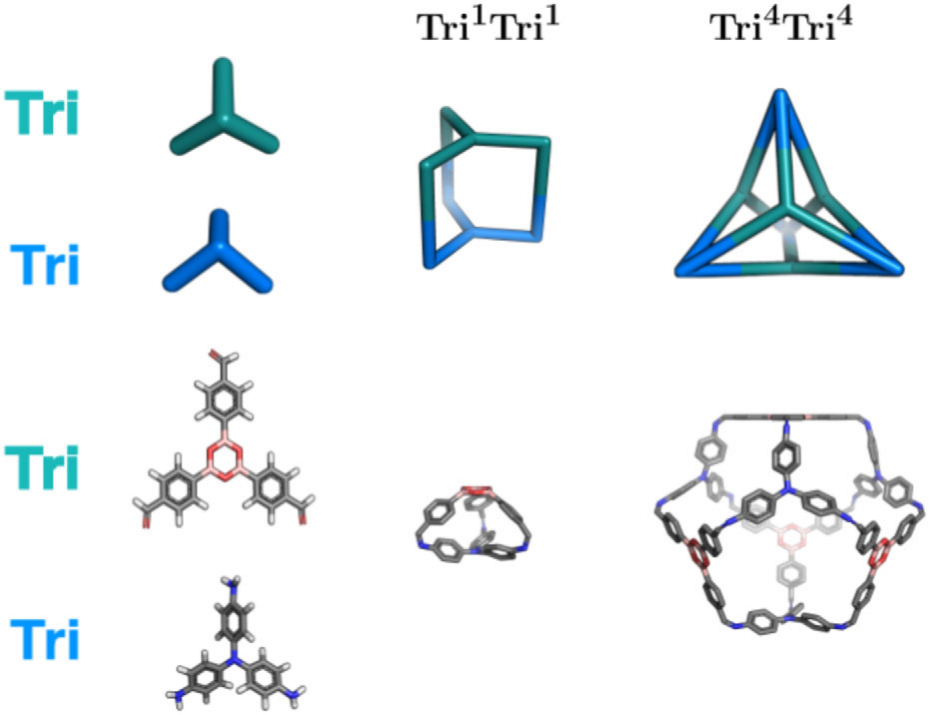
Cage topologies of the (**Tri + Tri**) family that can be constructed by *stk.* The molecular building blocks are shown on the left and for the topologies, schematics of the topologies are shown above examples constructed and optimized with *stk* from different numbers of the molecular building blocks. Hydrogen atoms are not shown in the topologies. [Color figure can be viewed at wileyonlinelibrary.com]

**Figure 7:**
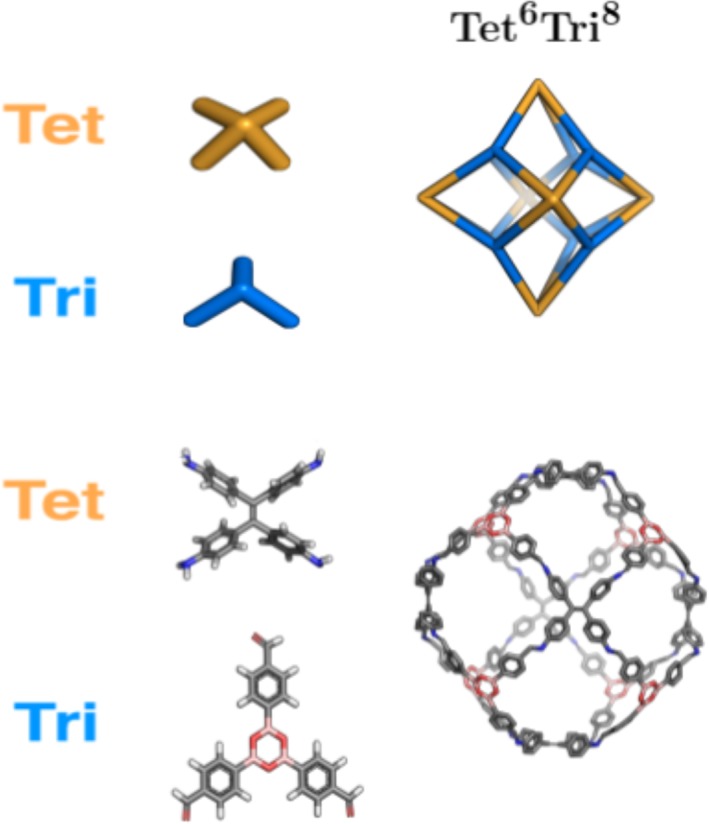
Cage topology of the (**Tet + Tri**) family that can be constructed by *stk.* The molecular building blocks are shown on the left, schematic of the topology above an example constructed and optimized with *stk* from the molecular building blocks. Hydrogen atoms are not shown in the topologies. [Color figure can be viewed at wileyonlinelibrary.com]

Due to the complex nature of predicting whether an organic cage will form from a set of monomers, discovery of these materials has been relatively slow. The assembly of these materials, even for computational studies, is a time consuming process. This is due to the relative complexity and diversity of their topologies, as well as the large number of possible structural isomers some cages can form. As a result, they are an ideal candidate for demonstrating the effectiveness of *stk* as a simple, general molecular assembly tool. While porous organic cages have many diverse topologies, they can be divided into four families, which are determined by the number of reactive end groups in the selected building block pair. In each family, construction in *stk* is implemented slightly differently, however the user input required to construct any cage is always the same, as only the building blocks and name of the topology need to be provided.

#### Construction

**Figure 8:**
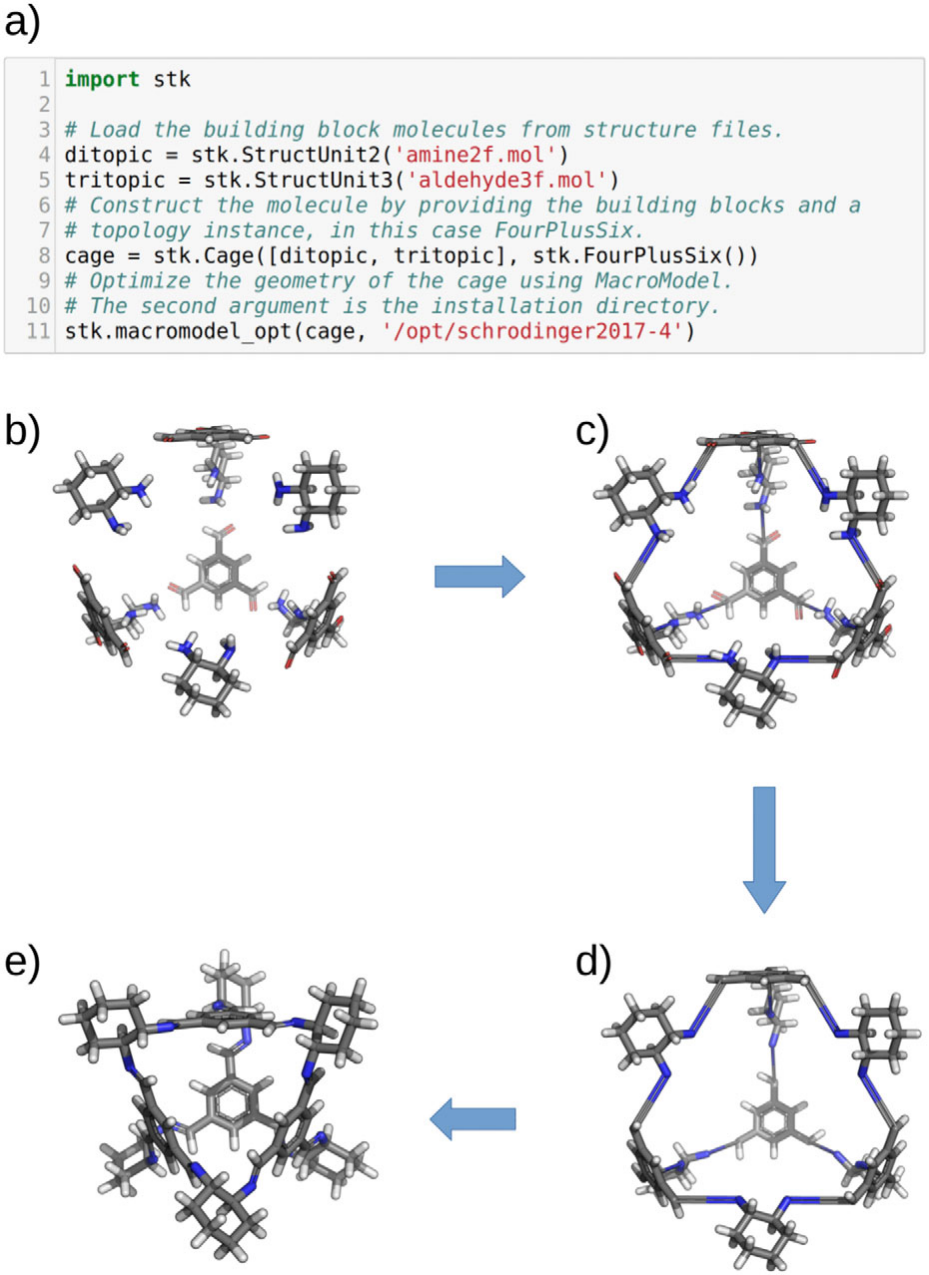
The assembly process of *stk*, and code necessary for construction, performed on a **Tri^4^Di^6^** cage with tetrahedral topology. a) Annotated code used to construct and optimize the cage molecule. b) The building blocks are placed and aligned on the vertices and edges of the topology. c) Bonds are formed between the bonder atoms. d) Redundant atoms are removed. e) The assembled supramolecule is then geometry optimized to a chemically plausible geometry. [Color figure can be viewed at wileyonlinelibrary.com]

When dealing with cage assembly, *stk* has three groupings of topologies, the construction of each of which involves a slightly different procedure. The first grouping is for topologies that contain a di‐topic linker; the (**Tri + Di**) and (**Tet + Di**) families, Figures 4 and 5, respectively. These show both the topologies and the cages generated by *stk* from the same set of building blocks for each family. An example of the construction of a **Tri^4^Di^6^** cage from this grouping, both in the code and visually, is shown in Figure 8. First, the building block with the greater number of reactive end groups (either tri‐topic or tetra‐topic) is placed on the vertices of the topology, while the di‐topic building block is placed on the edges (Fig. 8b). To support the creation of every possible structural isomer of a cage, a number of optional parameters can be provided by the user when initializing a **Topology** object. For each building block placed at a vertex, the user can specify which bonder atom is to bond with a di‐topic linker on which edge (there will be three connection options for a tri‐topic building block and four for a tetra‐topic building block). The building block will then be rotated so that the distance between the two atoms to be bonded is minimized. The remaining bonder atoms then each bond to the di‐topic linkers on their nearest edges (Fig. 8c) and the deleter atoms are removed (Fig. 8d). In addition, each building block placed on an edge can be aligned either parallel or anti‐parallel with it, as using asymmetrical ditopic building blocks will lead to a different connectivity in each case.

The second grouping of cage topologies is the (**Tri + Tri**) family, which involves two tri‐topic building blocks (which can be distinct or identical in *stk*). These topologies are shown in Figure 6, including examples of molecules constructed with *stk.* For this group, the two tri‐topic building blocks are placed on vertices and directly connected without any building block on the “edge” of the topology. Finally, the third grouping of cages is the **Tet + Tri** family, which has only a single member, the **Tet^6^Tri^8^** topology, as shown in Figure 7. In this grouping, the tri‐topic and tetra‐topic building blocks are placed on alternate vertices.

**Figure 9:**
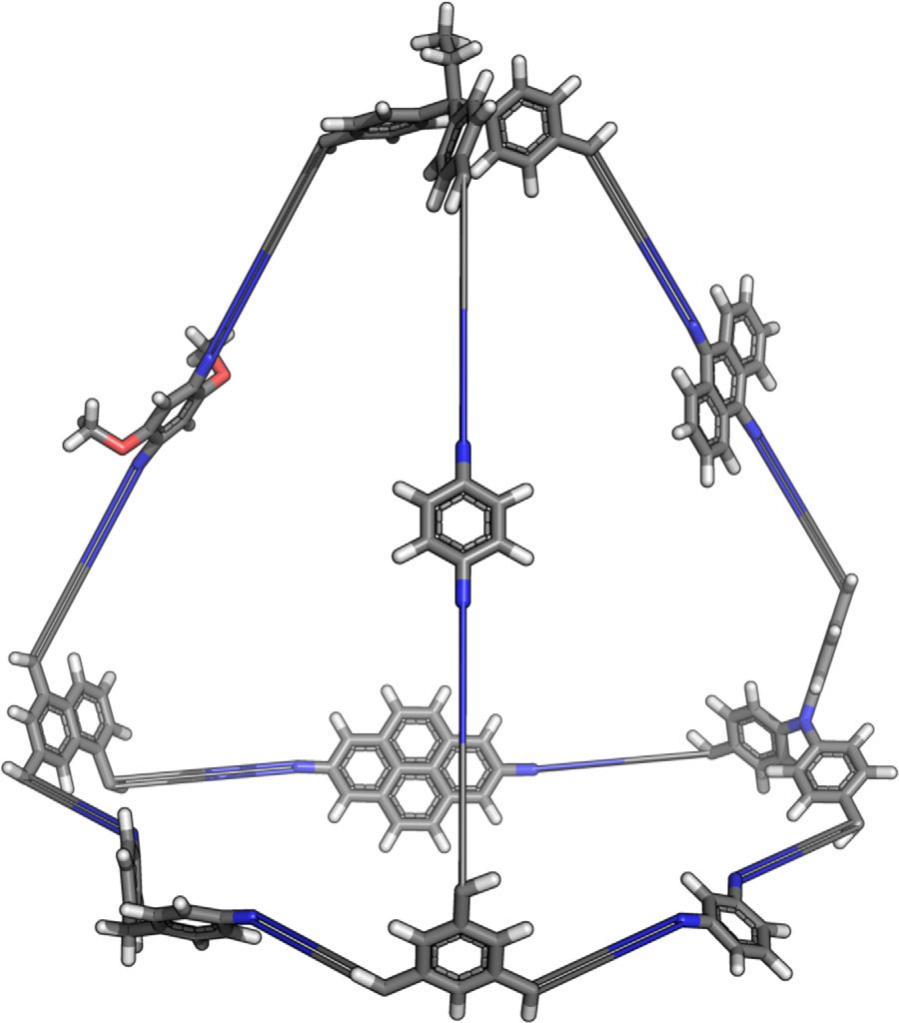
A multi‐component cage assembled by *stk* (prior to any geometry optimization). Each edge and vertex features a different building block, as an extreme case of a multi‐component cage. [Color figure can be viewed at wileyonlinelibrary.com]

In addition to structural isomers, all cage types support the construction of multi‐component cages. Multi‐component cages, for example those of Klotzbach and Beuerle,^44^ have more than two constituent building blocks assembled into a cage. These are constructed by simply adding more building blocks into the list provided to the **Cage** initializer. By default *stk* will select a building block with the correct topicity to place on a vertex or edge at random, however the user can also specify on which vertex or edge each building block is placed during initialization of the **Cage** object. An example of a multi‐component cage constructed by *stk* from 4 tritopic and 6 ditopic building blocks can be seen in Figure 9.

The **Cage** object produced after construction can be used for further studies in a number of ways. In addition to the properties mentioned earlier, *stk* allows users to directly calculate properties specific to cages, such as cavity and window size, the latter of which is calculated using the pyWindow library.^45^ For more in‐depth analysis of cage behavior, the assembled molecule, after optimization, can be used as input for MD calculations and further analysis, such as calculation of a pore limiting envelope^46^ or to simulate solvent effects.^47^ The molecule could also be used an input for crystal structure prediction, to predict the preferred solid state structure.^36^

### Linear polymers and small molecule oligomers

#### Context

Linear polymers are employed in a range of applications, which may be simple, such as food packaging, or complex, such as hydrogen evolution through photocatalytic water‐splitting,^48^, ^49^ organic electrochemical transistors^50^ and metal–ion batteries.^51^ Similarly, the great diversity of small oligomers leads to a number of applications in electronic devices.^52^, ^53^, ^54^ Predicting the potential of any of these materials for a given application is often a question of calculating and then balancing various trade‐offs. For example, the activity of polymeric photocatalysts is the result of a complex interplay of factors such as light absorption, band alignment and polymer wettability.^55^ However, because of the great diversity of monomers and vast number of possible combinations into polymeric and oligomeric materials, particularly once co‐polymers and beyond are considered, the phase space to explore for even linear polymers is enormous. It is therefore useful to be able to automate the construction of a large number of polymers and oligomers from monomer building blocks and to then evaluate their performance for a given application through computational screening. While tools for building polymers already exist 24, 25, 26, 27, 56, 57, 58 they are often specialized and work as standalone software. This means that integrating them into diverse workflows is more difficult and that experience using one tool cannot be used to build other supramolecular classes in the future. The advantage of our Python‐based implementation is that polymer assembly can easily be introduced into an existing modeling pipeline. Software development time is minimized due to Python's general simplicity and the absence of a compilation step.

#### Construction

**Figure 10:**
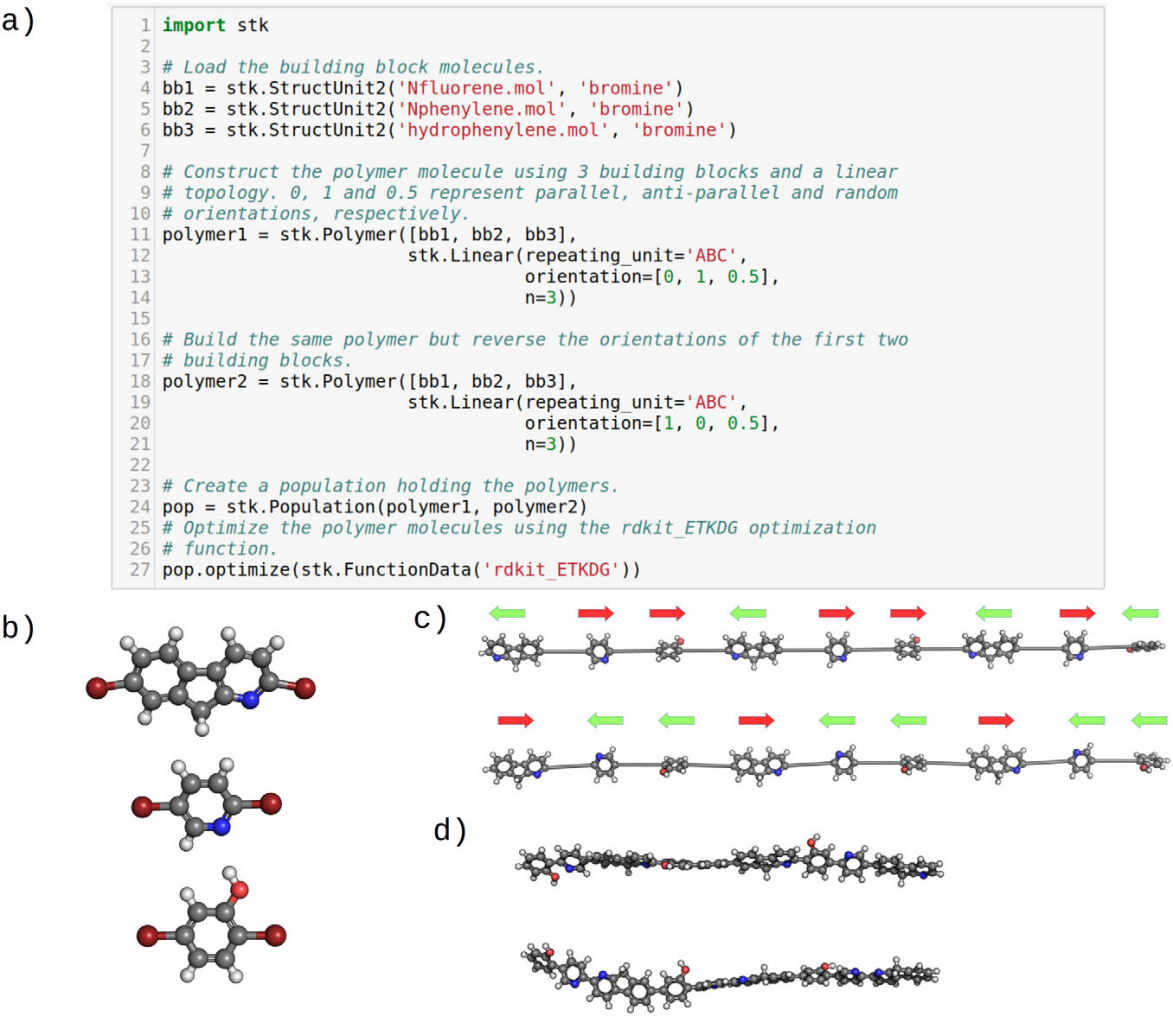
The assembly of a linear polymer by *stk.* a) The required code. b) The building blocks. c) The building blocks are placed on a straight line and connected with redundant atoms removed. The user can control the orientation of each monomer, shown with red and green arrows, the repeating unit and the number of repeating units joined. d) Geometry optimized polymer structures. [Color figure can be viewed at wileyonlinelibrary.com]

When considering the connectivity between building blocks, linear polymers are a relatively simple class of molecule from a structural perspective. For the construction of finite size linear polymer models in *stk*, only a list of building blocks corresponding to the monomers, a string identifying the polymer repeat unit and the number of repeat units is required as input. To construct the model, *stk* will use the repeat unit string to place the building blocks in a line in the correct order. As each building block encodes a bonder–bonder direction vector, they can be placed parallel or anti‐parallel to the chain. Alternatively, the choice can be made by *stk* at random at each point along the chain, where the probability of switching from parallel to anti‐parallel can be specified by the user for each monomer. This gives the user full control over the possible sequence isomerism of the polymer, in that a completely head‐to‐tail or completely head‐to‐head chain can assembled, as well as a chain with an arbitrary preference of head‐to‐head over head‐to‐tail. The latter is achieved by specifying a probability for a monomer to flip from parallel to anti‐parallel, as opposed to guaranteeing a parallel or anti‐parallel orientation in the first two cases. An example of the code and the resulting assembled polymer can be seen in Figure 10. The code also demonstrates how *stk* optimizes a population of objects.

The assembled polymer chain can be used as input for further calculations. Because *stk* provides great freedom in how the optimization is performed, how long the chain is, as well as its isomerism, the final structure can be tuned for a variety of studies. *stk* already allows users to calculate properties which may be useful when examining polymeric systems, such as the distance between the two furthest atoms in a polymer. Finally, because *stk* provides tools for translating and rotating molecules, a polymer chain can be used to prepare a multi‐chain simulation box, by repeatedly modifying the position of the chain.

**Figure 11:**
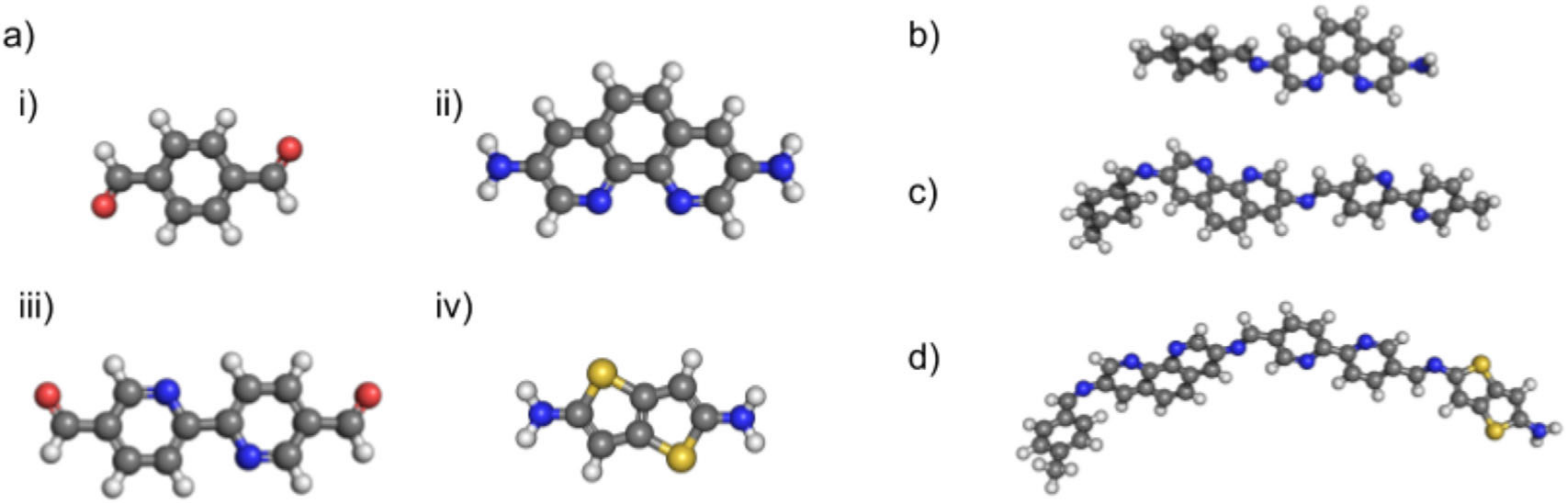
Small molecule oligomers assembled by *stk.* a) The building blocks (aldehydes and amines), which will be joined through an imine condensation reaction. b) Dimer constructed from i) and ii). c) Trimer constructed from i), ii) and iii). d) Tetramer constructed from i), ii), iii) and iv). [Color figure can be viewed at wileyonlinelibrary.com]

The polymer API can also be used to construct small molecules such as dimers and other oligomers. For dimers, the user needs to provide two building blocks and set the number of repeating units to 1, everything remains identical to the polymer case. For other oligomers, additional building blocks can be provided. These assemblies can be seen in Figure 11. When assembling small molecules using the **Polymer** class, *stk* creates new **Polymer** objects and as a result all methods available for these molecules can be used.

### Covalent organic frameworks (COFs)

#### Context

COFs are crystalline organic structures composed of carbon and other light elements such as boron, nitrogen, oxygen and silicon.^59^ These materials were first successfully synthesized by Côté et al. in a paper describing the production of a boron‐containing COFs.^60^ Since then, other varieties of COF have been developed, such as triazine^61^ and imine‐based COFs.^62^ COFs are typically low density with large surface areas, and can have good thermal and chemical stability. These features make them attractive for a range of applications, including gas storage and separation, drug delivery, catalysis, sensing and photoelectronic devices.^59^

COFs can be categorized into two broad types of topologies. The first, and most common, type are 2D COFs, which consist of stacked two‐dimensional, non‐covalently bonded sheets. These COFs have been synthesized in four distinct topologies: hexagonal, honeycomb, square and kagome, all of which have been implemented within *stk.* Second, there are 3D COFs, which feature a three‐dimensional network of covalent bonds, often via an sp^3^ carbon or silicon atom.^62^ COFs have been synthesized through a variety of reactions, however, the most common approach is to use reversible DCC reactions. Whilst these materials show much promise, to assist in the design of these materials, it is valuable to have open source software that can assemble both individual systems, for inspection by synthetic researchers, prior to attempted synthesis, as well as the ability to high‐throughput screen the systems for applications computationally. As a result, we hope that *stk* will be a useful tool for researchers interested in these materials.

#### Construction

**Figure 12:**
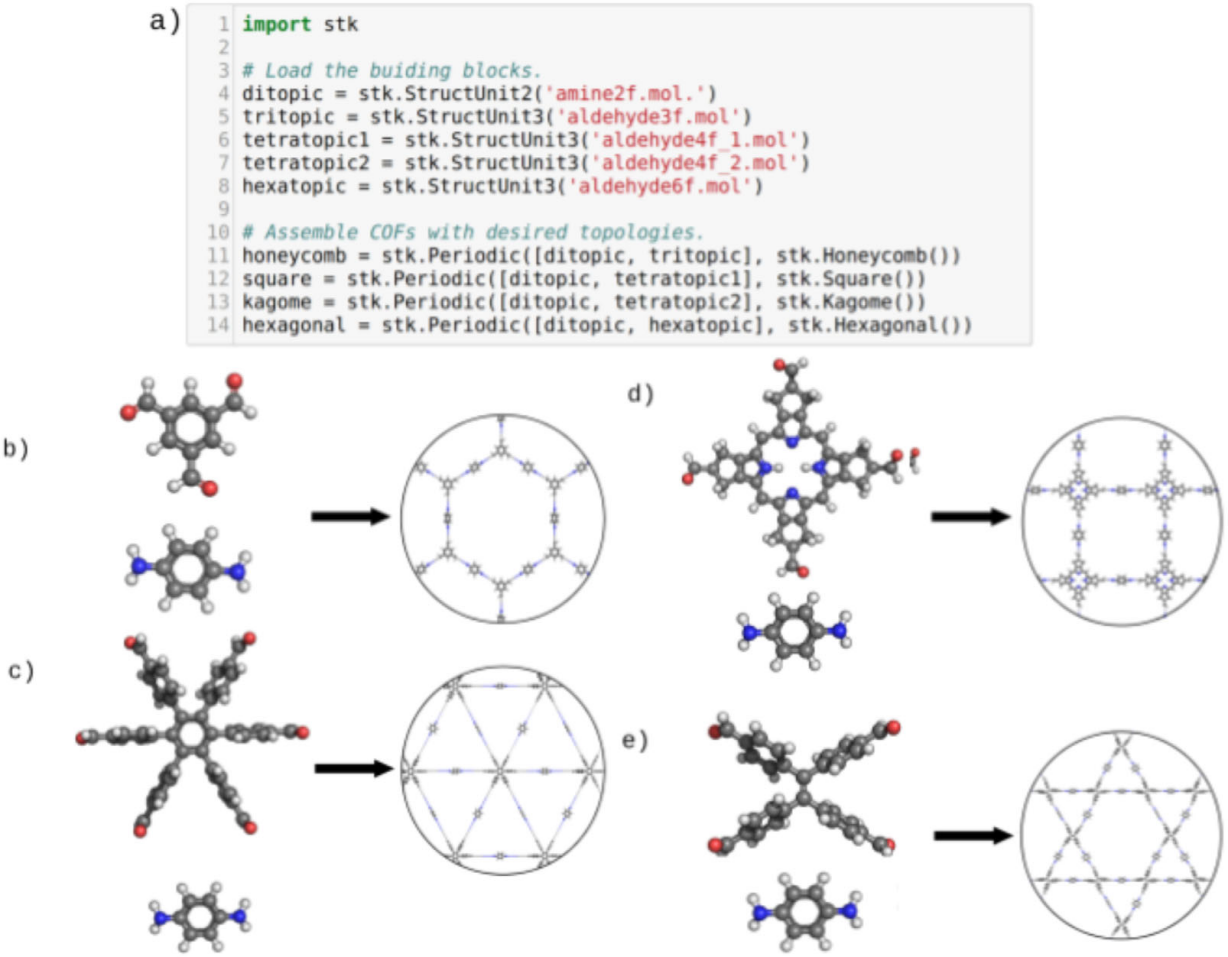
COF topologies assembled by *stk.* a) The code required to generate a periodic unit cell of the COFs shown in b)–e). The topologies are: b) honeycomb (*hcb*) c) hexagonal (*hxl*) d) square (*sql*) and e) Kagome (*kgm*); in each case the molecular building blocks are shown on the left and the periodic structure on the right. [Color figure can be viewed at wileyonlinelibrary.com]

The current implementation supports the construction of the four synthesized 2D‐COF topologies, but is easily extendable to other 3D topologies. The currently supported topologies are honeycomb, hexagonal, square and Kagome, with underlying net types of *hcb, hxl, sql* and *kgm*, respectively. Each of these topologies consists of two building blocks, one ditopic and one with three or more functional groups, referred to as multi‐topic in the following discussion. The multi‐topic building block in the hexagonal topology has three functional groups, the square and kagome building blocks have four functional groups and the hexagonal multi‐topic building block has six functional groups. The construction process for each of these topologies is identical, with only the number and connectivity of the vertices and edges changing. Examples of assembled COFs can be seen in Figure 12. A multi‐topic building block is placed on each vertex in the topology, while a di‐topic building block is placed on each edge. Each multi‐topic building block lies with its bonder atoms flat on the *xy* plane and can be rotated along the *z*‐axis to generate different structural isomers in cases where the building block is not symmetrical. Similarly, each di‐topic building block can be aligned parallel or anti‐parallel with the edge it is placed on. To join the building blocks, bonds are created between bonder atoms on the di‐topic building blocks with the bonder atoms on the multi‐topic building blocks. The created bonds may be direct or periodic, depending on which building blocks are being joined.

Due to the construction process placing building block molecules on an idealized geometry, represented by the vertices and edges of a perfect unit cell, the bonds added during assembly will have unrealistic lengths. To correct this and find a realistic structure an optimization and conformer search should be performed using an optimization function. The constructed COF model can be written as a periodic unit cell into a GULP^63^ input file and this can be extended to other periodic codes. *stk* further provides the option for producing molecular “islands” of arbitrary size. Molecular islands are finite size cut outs of the periodic material, with terminating atoms used in place of periodic bonds. Future extensions to *stk* can include the calculation of COF features such as pore size and surface area, or to run calculations such as Grand Canonical Monte Carlo simulations to determine gas sorption properties.

## Conclusions

*stk* is a Python library designed to assemble, optimize and calculate the properties of supramolecules. It aims to provide a modular, extensible framework that can be applied to a range of different supramolecules. In general, during the assembly process building block molecules are placed onto a predefined topology and bonds are formed between them to generate a supramolecule. Currently supported molecules include linear polymers, small molecule oligomers, organic cages and COFs. Furthermore, *stk* allows a range of calculations, which can be performed with molecules loaded directly into the library or those assembled by it. The library provides tools to use these molecules in input files for further calculations and in high‐throughput screening. In the future, we believe that *stk* will be extended to other complex materials such as metal organic frameworks, metallocycles and rotaxanes.
